# Acoustic Communication and Sound Degradation: How Do the Individual Signatures of Male and Female Zebra Finch Calls Transmit over Distance?

**DOI:** 10.1371/journal.pone.0102842

**Published:** 2014-07-25

**Authors:** Solveig C. Mouterde, Frédéric E. Theunissen, Julie E. Elie, Clémentine Vignal, Nicolas Mathevon

**Affiliations:** 1 Equipe de Neuro-Ethologie Sensorielle ENES-CNPS CNRS UMR8195, Université de Lyon/Saint-Etienne, Saint-Etienne, France; 2 Helen Wills Neuroscience Institute, University of California, Berkeley, California, United States of America; Claremont Colleges, United States of America

## Abstract

**Background:**

Assessing the active space of the various types of information encoded by songbirds' vocalizations is important to address questions related to species ecology (e.g. spacing of individuals), as well as social behavior (e.g. territorial and/or mating strategies). Up to now, most of the previous studies have investigated the degradation of species-specific related information (species identity), and there is a gap of knowledge of how finer-grained information (e.g. individual identity) can transmit through the environment. Here we studied how the individual signature coded in the zebra finch long distance contact call degrades with propagation.

**Methodology:**

We performed sound transmission experiments of zebra finches' distance calls at various propagation distances. The propagated calls were analyzed using discriminant function analyses on a set of analytical parameters describing separately the spectral and temporal envelopes, as well as on a complete spectrographic representation of the signals.

**Results/Conclusion:**

We found that individual signature is remarkably resistant to propagation as caller identity can be recovered even at distances greater than a hundred meters. Male calls show stronger discriminability at long distances than female calls, and this difference can be explained by the more pronounced frequency modulation found in their calls. In both sexes, individual information is carried redundantly using multiple acoustical features. Interestingly, features providing the highest discrimination at short distances are not the same ones that provide the highest discrimination at long distances.

## Introduction

Birds' acoustic signals transmitted over large distances degrade in amplitude and in spectral and temporal structure as they propagate through the environment [1], [2]. These propagation-induced degradations reduce the active space of the signal, i.e. the distance from the emitter over which the information can be decoded by a receiver [3]–[6]. One of the challenges in songbird vocal communication is to investigate this acoustic active space, and more specifically to understand how degradation affects the message emitted by the sender as well as the response of the receiver(s).

Among various pieces of information that birds' vocalizations can potentially encode, cues about individual identity (individual signature) are particularly important. Indeed, individual recognition plays a fundamental role in male/female communication in the contexts of courtship behaviors and pair bond maintenance (especially in monogamous species) [7]–[9], as well as for territorial birds that need to recognize the vocalizations of strangers from neighbors in order to react accordingly [10], [11]. Moreover, individual recognition between parent and offspring is critical for breeding success, especially for colonial species that do not use fixed nest sites [12], [13]. The preservation of individual signature in propagated sounds raises an interesting question as it appears that individuality requires fine temporal and spectral information that may be highly susceptible to propagation-induced degradation [14].

To our knowledge however, only two studies providing an acoustic analysis of the long-range degradation of the individual signature in songbirds have been published. In the white-browed warbler *Basileuterus leucoblepharus*, the territorial song of the male consists of a succession of pure tones slowly decreasing in frequency. The emitter's individual identity is encoded in the first half of the song, which is high-pitched. After long range transmission, this first half, and consequently the individual signature, disappears due to the great susceptibility of high frequencies to degradation through the forest environment [14], [15]. Conversely, in the territorial call of the male corncrake *Crex crex*, the acoustic feature that is the most characteristic of each individual is the inter-pulse duration. This temporal code remains practically constant after propagating 100 m through dense vegetation, making it a good candidate for encoding the vocal signature at a distance [16].

In these two previous studies, the individual identity is coded either by frequency modulation of pure tones (white-browed warbler) or inter-pulse duration (corncrake), which makes the assessment of propagation-induced modifications quite simple. In the case of the white-browed warbler, the frequency-dependent attenuation over distance is responsible for the disappearance of the high-pitched notes, and for the corncrake the filling of inter-pulse silences by echoes is the main factor explaining the progressive loss of individual information. Conversely, the quantification of propagation-induced information loss of complex sounds displaying large frequency bandwidths together with amplitude and frequency modulations could be more problematic. In the present study, we focused on the distance calls of the zebra finch *Taeniopygia guttata*, which fit these characteristics.

**Figure 1:**
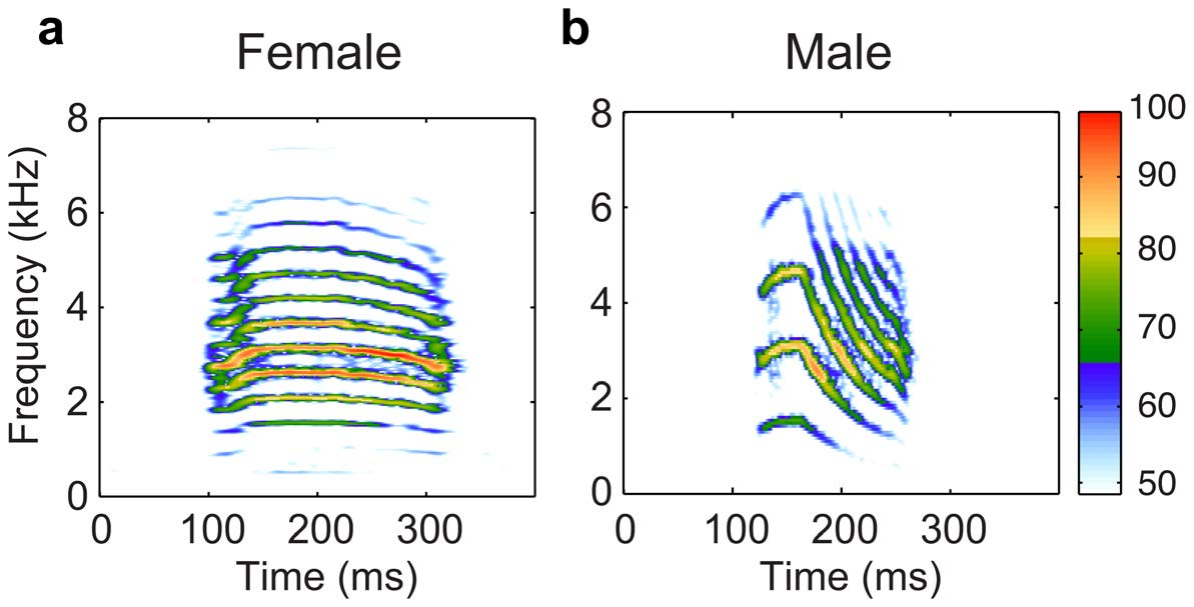
Spectrograms of a female (a) and male (b) distance call, recorded at 2 m. Both signals were high-pass filtered above 500 Hz to avoid displaying low-frequency background noise. The color scale is in relative dB as shown on the color bar with 100 dB corresponding to maximum amplitude observed.

Zebra finches are small gregarious songbirds from subarid regions of Australia which form strong pair bonds for life and live in large flocks in open grassy country with a scattering of trees and bushes [17], [18]. They use different types of vocalizations, one of the most frequently heard being the distance call. This loud call is emitted by both sexes and is used when birds establish acoustic contact at a distance [18]. It is a complex sound, consisting of a harmonic series modulated in frequency as well as amplitude. Distance calls differ between males and females [19], [20], the males' fundamental frequency being higher than the females' (typically 650-1000 Hz versus 500-600 Hz) as well as usually being shorter and more frequency-modulated (Fig. 1). It has been shown that the distance call bears an individual signature [21], and that zebra finches are capable of call-based individual recognition [8], [9], [19], [22], [23]. The “active space” of these calls has been estimated based on naturalistic observations to be up to 100 meters [18]; similar conclusions were reached with theoretical calculations using discrimination thresholds for masked non-degraded signals in this species [4]. However, no experimental work has yet investigated the long-range transmission of the individual signature in the distance call.

Here we characterized and quantified the degradation of female and male zebra finch calls during propagation, focusing on the acoustical features that carry information about individual identity. We performed this descriptive analysis to answer three sets of questions. First, what are the acoustical features that carry the most information about the individual signature, and are these most informative features the same at different distances? Second, how would a discrimination performance be affected if the animal based its discrimination on a single and distance independent set of acoustical features instead of using different sets of features adapted to each tested distance? Finally, are there significant differences in the individual signature of female vs. male calls in the way they degrade with propagation?

## Materials and Methods

We performed two sets of propagation measurements: the first set was performed in Australia in a typical environment of the wild zebra finch, and the second set was performed in France in an open field. The measurements in Australia were performed with a limited database of zebra finch calls and can be considered as a pilot study for characterizing the acoustic properties of the species' natural environment. The background noise distributions and the frequency transfer functions obtained in both environments were compared to assess both the ecological relevance and some of the limitations of these analyses. The propagation experiments performed in France included multiple call exemplars of each individual, and aimed to fully characterize the acoustic features that carried information about the caller's identity.

### Ethics statement

The experimental protocol for the recording of distance calls of our zebra finch subjects was approved by the Jean Monnet University's animal care committee (authorization n°42-218-0901-38 SV 09 to the ENES lab).

### Calls database used in the experiments and recording locations

For the pilot experiments performed in Australia, we used distance calls from both wild and domesticated zebra finches (16 calls used: 2 samples each from 2 wild males, 2 wild females, 2 domesticated males and 2 domesticated females). The domesticated zebra finches had been previously recorded in France (see protocol below). The distance calls from the wild birds were recorded at the University of New South Wales Arid Zone Research Station at Fowlers Gap, 112 km north of Broken Hill in far western New South Wales, Australia (Gap Hills; 30°57′S, 141°46′E). Here, a large zebra finch colony is established, with birds breeding both in natural vegetation and in nest-boxes [24]. We recorded calls using omnidirectional tie-microphones (AKG C417) placed inside nest-boxes and connected to a portable solid state recorder (Marantz PMD670; sampling frequency: 44100 Hz; D&M Holdings Inc., Kanagawa, Japan). These recordings were conducted under the authorities of the Animal Ethics Committees at the University of New South Wales and Macquarie University and a Scientific Research Permit from the New South Wales Parks and Wildlife Service.

For the propagation measurements performed in France, we used calls from 32 domesticated zebra finches (16 distance calls from each of 16 females and 16 males  = 512 calls). The domesticated birds were bred in the ENES laboratory aviary (14L/10D photoperiod with adapted wavelengths; food and water *ad libitum*; temperature between 23 and 25°C). They were recorded in a soundproof room using a Sennheiser MD-42 microphone placed 0.2 m above the cage and connected to a Marantz PMD 670 recorder (sampling frequency: 44100 Hz). Each bird was recorded in the presence of two female zebra finches placed 3 m away and used as an audience to minimize stress [25]; the bird was stimulated with playbacks of distance calls that had previously been recorded from conspecific birds. During the recording, conditions of temperature, food and water availability were the same as in the aviary. All the calls were normalized by matching the maximum values of the sound pressure waveforms using Goldwave (version 5.57), prior to the propagation experiments. The frequency power spectra of wild and domesticated zebra finches were similar: we did not observe any consistent differences that could have been due to our use of different recordings methods or to different vocal outputs of the two populations. The two types of vocalizations were thus combined in our analysis of the pilot experiment in Australia.

### Recording and processing of propagated calls

#### 1. Sound transmission experiments in Australia

The transmission experiments in Australia were performed in November 2008 at two locations, next to the research station. The first experimental location was an open environment deprived of vegetation. On this site, experiments were performed during the day with light wind. The weather was clear and the temperature was around 30°C (Australia site 1). The second location was a subarid environment with sparse vegetation including small trees, the landscape being open with little relief [18]. Experiments at this location were performed on a completely windless evening. The weather was clear and the temperature was around 25°C (Australia site 2).

For both Australian recordings, the sounds were played back via an amplified loudspeaker (Minivox Lite PA System, Anchor Audio, Inc.) connected directly to a laptop computer. The loudspeaker was placed at a height of between 1 and 1.3 m. The volume of the speaker was set such as to match the intensity typical of zebra finch calls, around 70 dB at 1 m [9]. Sounds were recorded using an omni-directional microphone (Sennheiser MD 42) connected to a Marantz PMD670 recorder (sampling frequency: 44100 Hz). The microphone was held at 1 m above the ground. We recorded the calls sequences 2 m, 5 m, 10 m, 20 m, 50 m and 100 m away from the speaker.

#### 2. Sound transmission experiments in France

The transmission experiments in France were performed in October 2010 in the afternoon on a level field in Bellegarde-en-Forez, Loire, France. This recording site was chosen to match the open environment of the Australian desert. The weather was cloudy, there was little wind and the temperature was 11°C. The complete calls database was broadcast from a portable solid state recorder (Marantz PMD671) connected to a MegaVox speaker (PB-35W) placed on a stool, 1.3 m high. The speaker volume was set to obtain a sound level of 70 dB SPL at 1 m (Velleman Sound Level Meter DVM 1326). The sounds were recorded with a Schoeps microphone (MK4 cardioid, on a CMC6-U base) equipped with a Schoeps Basket-type Windscreen (W 20) and set 1.3 m high. The microphone was connected to a second Marantz recorder (PMD671; sampling frequency: 44100 Hz). We recorded the calls 2 m, 16 m, 64 m, 128 m and 256 m away from the source, twice for each distance.

#### 3. Processing of propagated signals

We used a custom-made Matlab (Mathworks) script to cross-correlate the propagated recordings with the original sequences. For the recordings performed in France, we then compared the signals of the propagated calls from the two recording sessions, by ear or by a visual assessment of spectrograms when necessary, and selected the better of the two recordings for each call and at each propagation distance. This selection allowed us to exclude recordings that had been impaired by unexpected transient sounds that were not relevant to the study (e. g. birds calling in the vicinity).

### Acoustic analysis

#### 1. Visualization of the spectro-temporal properties of the environmental noise and the propagated calls

##### a. Frequency spectra

Using custom-made Matlab scripts, we calculated the frequency spectra (FS, in dB units) for all calls from the same sex at each propagation distance. We also calculated the FS of the background environmental noise that was present during these recordings.

##### b. Spectral transfer function

Spectral transfer function can be used to quantify signal attenuation (or gain) and phase shifts as a function of frequency for a given propagation distance. Here we calculated, the gain of spectral transfer function by normalizing the cross-spectrum between a propagated sound and the reference sound by the FS of the reference sound. We used the recordings obtained at 2 m as the reference sounds, and the recordings at 50 m (Australia) and 64 m (France) as the second propagated sound. In other words, we estimated the gain of the transfer function resulting from 48 m of propagation in Australia and 62 m in France. Estimates of the standard error in the gain function were obtained using the Jackknife resampling technique [26]. The empirical transfer function was compared to the attenuation expected from spherical radiation and atmospheric absorption. The sound level of a source emitting at a sound level L_s_ (in dB) and recorded at a propagation distance r, L_r_, can be written as:
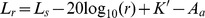
where the −20 log_10_(r) term corresponds to the spherical distribution of energy (also known as the inverse square law and yielding a 6 dB attenuation per distance doubling), K′ is a constant equal to −10 log_10_(4π) for spherical radiation and −10 log_10_(2π) for hemi-spherical radiation and A_a_ is the atmospheric absorption. Atmospheric absorption depends on frequency, temperature, humidity and atmospheric pressure and its magnitude can be estimated analytically using the equations published by the International Organization for Standardization (ISO 9613–1:1993). These equations were implemented in a Matlab script that was validated by comparing its output to the tables provided in the ISO publication. For example, at 1 atm, for 50% humidity and at 4 kHz, the atmospheric attenuation is 4.5 dB/100 m, 2.6 dB/100 m and 2.5 dB/100 m at 11°C, 25°C and 30°C, respectively.

##### c. Modulation power spectr

In order to visualize the joint spectro-temporal modulations of the distance calls propagated in France and their evolution with propagation, we calculated the modulation power spectra (MPS) of the calls: the joint second order statistics of the spectro-temporal amplitude envelopes obtained from a spectrographic representation of the sound [27]. The MPS is obtained as follows. We first calculated the spectrogram of each call using a Gaussian window (symmetric in time and frequency domains) 70 Hz wide in the frequency domain or 2.27 ms wide in the time domain. The MPS is then simply the 2-D power spectrum of the log spectrogram [27]. The time-frequency scale of the spectrogram (70 Hz) determines the spectral and temporal Nyquist limits of the modulation spectrum (7.14 cycles/kHz for spectral modulations and 220 Hz for temporal modulations). MPS were obtained for each propagation distance and each sex.

#### 2. Parameters used for the statistical analysis of individual signature

We used two distinct sets of acoustic parameters to test for the presence of a vocal signature in the propagated calls: (a) a set of parameters that separately describe the amplitude in the spectral domain (the spectral envelope) and the amplitude in the time domain (the temporal envelope, often simply called amplitude envelope); (b) the spectrogram. Since our spectrographic representations are invertible, using a complete spectrogram not only circumvents the use of *a priori* assumptions on the nature of the information-bearing acoustical features but also provides an upper bound for discriminability.

Prior to these analyses, the sounds were band-pass filtered between 0.5 to 8 kHz in order to reduce irrelevant environmental background noise. Those frequency cutoffs were chosen based on the zebra finch's audiogram [28].

##### a. Parameters used to describe the separate spectral and temporal features

We extracted the spectral amplitude envelope (amplitude as a function of frequency) and temporal amplitude envelope (amplitude as a function of time) of each call. Each amplitude envelope (spectral and temporal) was then converted to a density function by dividing each value of amplitude by the sum of all amplitude values. We quantified the shape of these normalized envelopes by estimating the moments of the corresponding density functions: their mean (i.e. the spectral centroid for the spectral envelope and temporal centroid for the temporal envelope), standard deviation (i.e. spectral bandwidth and temporal duration), skewness (i.e. measure of the asymmetry in the shape of the amplitude envelopes), kurtosis (i.e. the peakedness in the shape of the envelope) and entropy. The entropy captures the overall variability in the envelope; for a given standard deviation, higher entropy values are obtained for more uniform amplitude envelopes (e.g. noise-like broad band sound and steady temporal envelopes) and lower entropy values for amplitude envelopes with high amplitudes concentrated at fewer spectral or temporal points (e.g. harmonic stacks or temporal envelope with very fast attack and decay). The spectral envelope was obtained with the Welch's averaged, modified periodogram estimation of the power spectral density using a Hann window of 23 ms and an overlap of 99%. The temporal envelope was obtained by rectifying the sound pressure waveform and low-pass filtering below 50 Hz. With these procedures, we obtained 10 acoustical parameters, 5 describing spectral features and 5 describing temporal features. Since these parameters had different units, Z-scores were calculated prior to using them in the multivariate discriminant analyses.

##### b. Full spectrographic representation

As stated above, we calculated an invertible spectrogram of each call using a Gaussian window and a time-frequency scale of 70 Hz-2.27 ms. Because the dimensionality of this representation was higher than the total number of calls in our database, we used a Principal Component Analysis (PCA; using the princomp function of Matlab) for dimensionality reduction. The discriminant analysis was then performed using the coefficients of a subset of the principal components.

**Figure 2:**
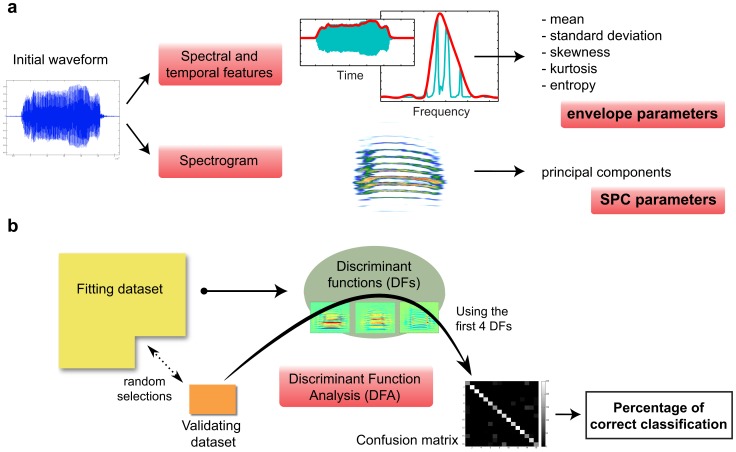
Overview of the acoustical analysis. This figure summarizes the procedure for extracting both sets of parameters (a), and the subsequent discriminant function analysis (b). The discriminant functions were calculated using the fitting dataset, and we tested the validating dataset against these to obtain the percentages of correct classification.

These two complementary approaches enabled us to compare the acoustical nature of vocal signatures, using discrete and more easily interpretable envelope parameters that have proven to be useful, if somewhat subjective, in the investigation of information-bearing features [29], as well as more complex parameters (principal components coefficients) extracted from a complete and invertible representation of the signals. To be concise, we will refer henceforth to the 5 pairs of separate spectral and temporal parameters as the “envelope parameters” and to parameters describing the spectrogram as the “spectrogram principal component parameters”, or more simply “SPC parameters”. The extraction method for each set of parameters is summarized in Fig. 2a.

#### 3. Statistical analysis of the vocal signature in calls

To test for the presence of an acoustic signature in the propagated distance calls, we used cross-validated linear discriminant function analyses (DFA), performed separately for each sex. The DFA finds the linear combinations of parameters that maximally separate the data points (the calls) that belong to different categories (the birds identities); these linear combinations are called discriminant functions (DFs) and the significant DFs define a Euclidian subspace where calls can be projected. The cross-validation consists of first calculating a set of discriminant functions using a fitting dataset, and then testing these functions on a validating dataset (non-overlapping with the fitting dataset) by assessing the number of calls that had been correctly classified for each individual, the percentage of correct classification (Fig. 2b). We performed this DFA using either the 10 envelope parameters or the SPC parameters.

Prior to the final calculations, we performed a series of preliminary analyses to determine the number of principal components (PCs) used for the SPC parameters and the number of discriminant functions (DFs) used for quantifying the discrimination for both the envelope and SPC parameters. We found optimal values for these hyper-parameters using the data at 2 m as a reference, and applied them for all other conditions. We first aimed to define the number of PCs to be used to reduce the dimensionality of the data while representing a reasonable amount of variance; to do this we estimated the percentages of correct classification using the 10 first significant DFs (at 2 m using the SPC parameters, 13 were significant for the females, and 15 were significant for the males as assessed by the Wilks Lambda test statistic) and a varying number of PCs from 10 to 100. We found that using 25 PCs captured most of the information about identity as witnessed by a saturation of the percentages of correct classification around that number (94.9% with 25 PCs and 95.4% with 100 PCs, using 10 DFs). We then compared the eigenvalues for each DF (fixing the number of PCs to 25) and found that using the first 4 DFs (DF1 to DF4) was an appropriate tradeoff, as it accounted for 83.9% for the females and 82.6% for the males of the variance between birds. Furthermore, in all our discriminant analyses (described below), we found at least 4 significant DFs. We were thus able to always use the same number of DFs in all our calculations and directly compare results. Finally, we assessed the number of cross-validation iterations required to obtain robust values for the percentage of correct classification; we found that 250 iterations were largely sufficient to reach stable values.

We quantified the discriminability by calculating the percentage of correct classification. We also determined which acoustical features were the most important for individual discrimination by examining the form of the significant DFs (for the SPC parameters) or the effect of each set of parameters independently (for the envelope parameters). The discriminant analyses were only performed for the more complete data set of propagated calls obtained in the experiments in France.

#### 4. Extraction of the most important parameters for individual discrimination

A different procedure was used for identifying the relative importance of the envelope parameters and the SPC parameters in the individual discrimination task. For the envelope parameters, we simply repeated the DFA with only the temporal envelope parameters or only the spectral envelope parameters. For the SPC parameters, we represented the first 4 DFs in the spectrographic space: since both the discriminant analysis and the PCA decomposition of the spectrogram are linear operations, the inverse rotation and scaling can be applied on the discriminant functions to represent them in a spectrographic space, allowing us to describe them as we would on a spectrogram.

#### 5. Comparisons between the percentages of correct classification

To compare the results obtained with different set of parameters, at different distances and for both sexes, we performed analyses of covariance (ANCOVA), using a general linear model framework (Statistical Toolbox in Matlab), with the percentage of correct classification as the dependent variable, the sex (male vs. female) and the type of parameters used (SPC vs. envelope) as factors, and the propagation distance as a covariate.

## Results

In a first step, we will describe the frequency dependent spectral changes in Australia and in France. In a second step, we will examine the changes in the temporal-spectral modulations due to propagation. In the remaining sections, we will then examine the structure in the calls that carries the information about identity, how this information changes for propagated calls, and whether these changes can be explained by the degradations.

### Spectral degradation in propagated signals in France and Australia

**Figure 3:**
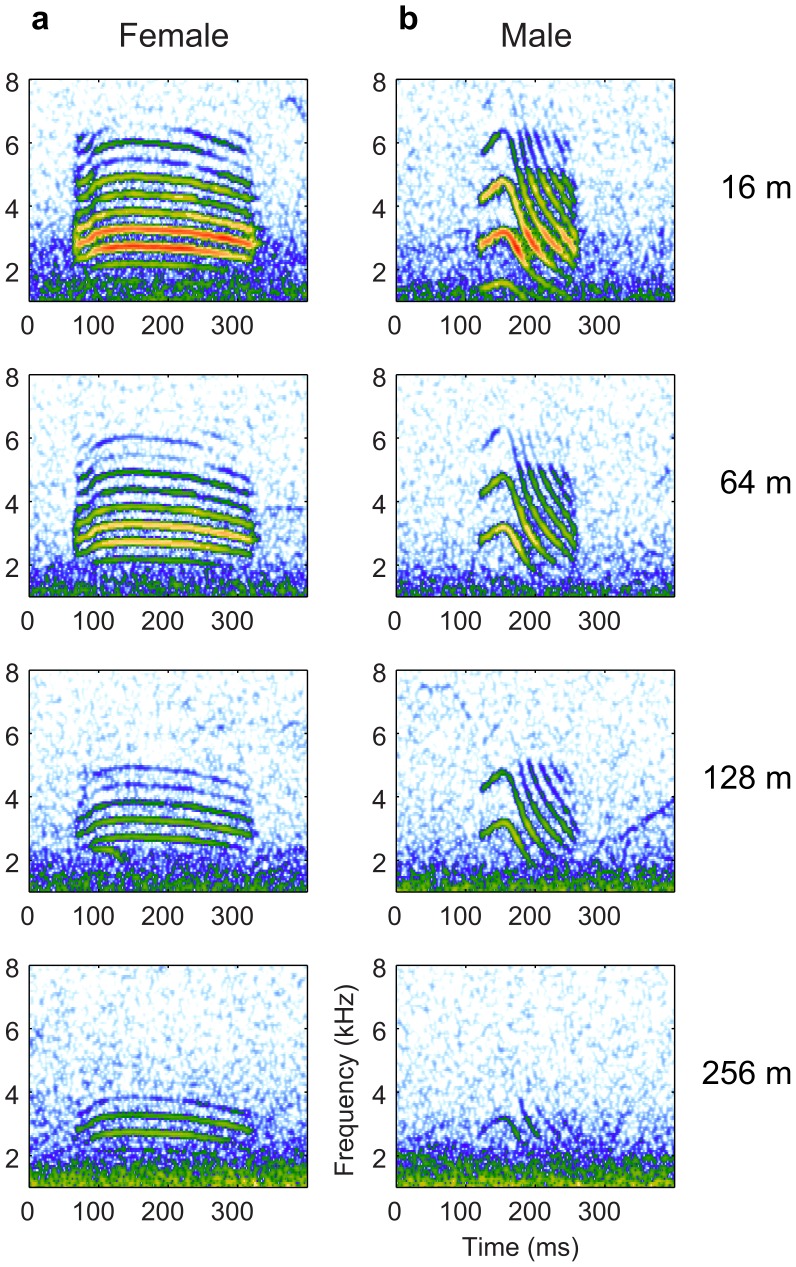
Spectrograms of the same female (a) and male (b) distance call recorded at various distances. All signals were high-pass filtered over 500 Hz, and the same color scale was applied to all spectrograms.

**Figure 4:**
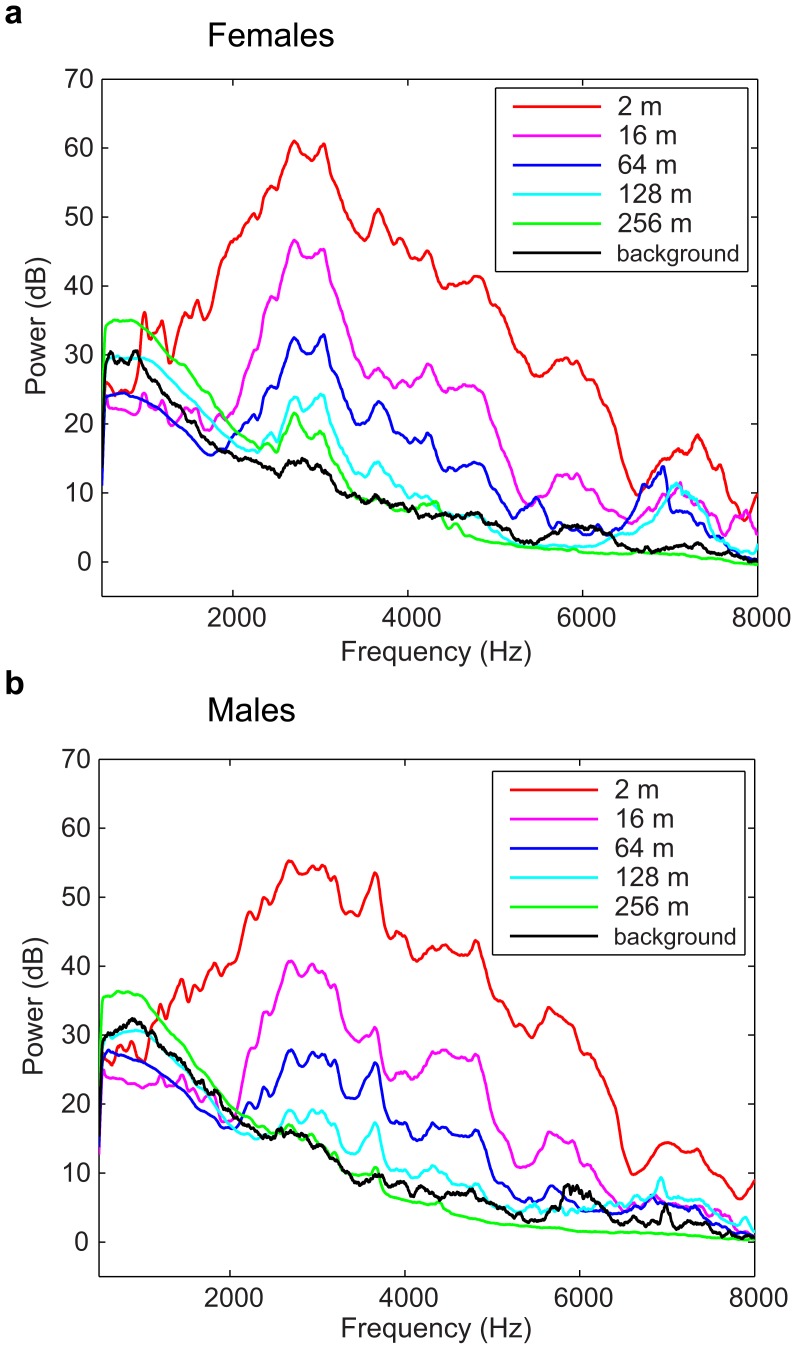
Frequency power spectra of calls at every propagation distance (from 2 m to 256 m). These were calculated using all female (a) and male (b) distance calls recorded in France. The frequency spectrum of the background noise at the recording sites is shown in black. This curve was obtained by averaging the noise spectrum across all recording sites and can therefore be slightly higher than the noise floor at particular distances, as it can be observed for the lower frequency range.

On Fig. 3, we illustrate the effect of propagation on signal quality by showing the spectrograms of the same female (a) and male (b) calls recorded at 16 m, 64 m, 128 m and 256 m in the open field in France (see also Audio File S1). The propagation-induced band-pass filtering of the signal is clearly shown, as well as the significant reduction of the signal-to-noise ratio (SNR) with distance. Specific higher-order spectral structures, such as the frequency structure in harmonic stacks of female calls that leads to a pitch percept, become also less salient as the fundamental drops below the noise level at low frequencies and as fewer harmonic components are available at higher frequencies. Similarly higher order temporal structures, such as the relative durations of the upsweep and downsweep in the male call, are also affected. Fig. 4 shows the mean frequency spectra (FS) of propagated calls for both females and males. The curves quantify the attenuation of the signal with increasing distance. The band-pass profile of the calls is clearly visible at 2 m, with a large peak centered around 3 kHz for both sexes. However, while the signal is clearly above the noise floor up to 8 kHz at 2 m, this upper frequency bound is reduced to 6 kHz at 64 m and around 4.4kHz at 128 m. Similarly, the lower frequency bound increases as a function of propagation distance. At further distances, signal energy is only found in a narrow peak centered at 3 kHz. In summary, the spectral degradation of propagated calls in France is characterized by a systematic reduction of the SNR at all frequencies and, given the profile of the power spectrum of the signal and noise, the region with a SNR >1 shrinks to a narrow-band centered around 3 kHz as propagation distance increases.

**Figure 5:**
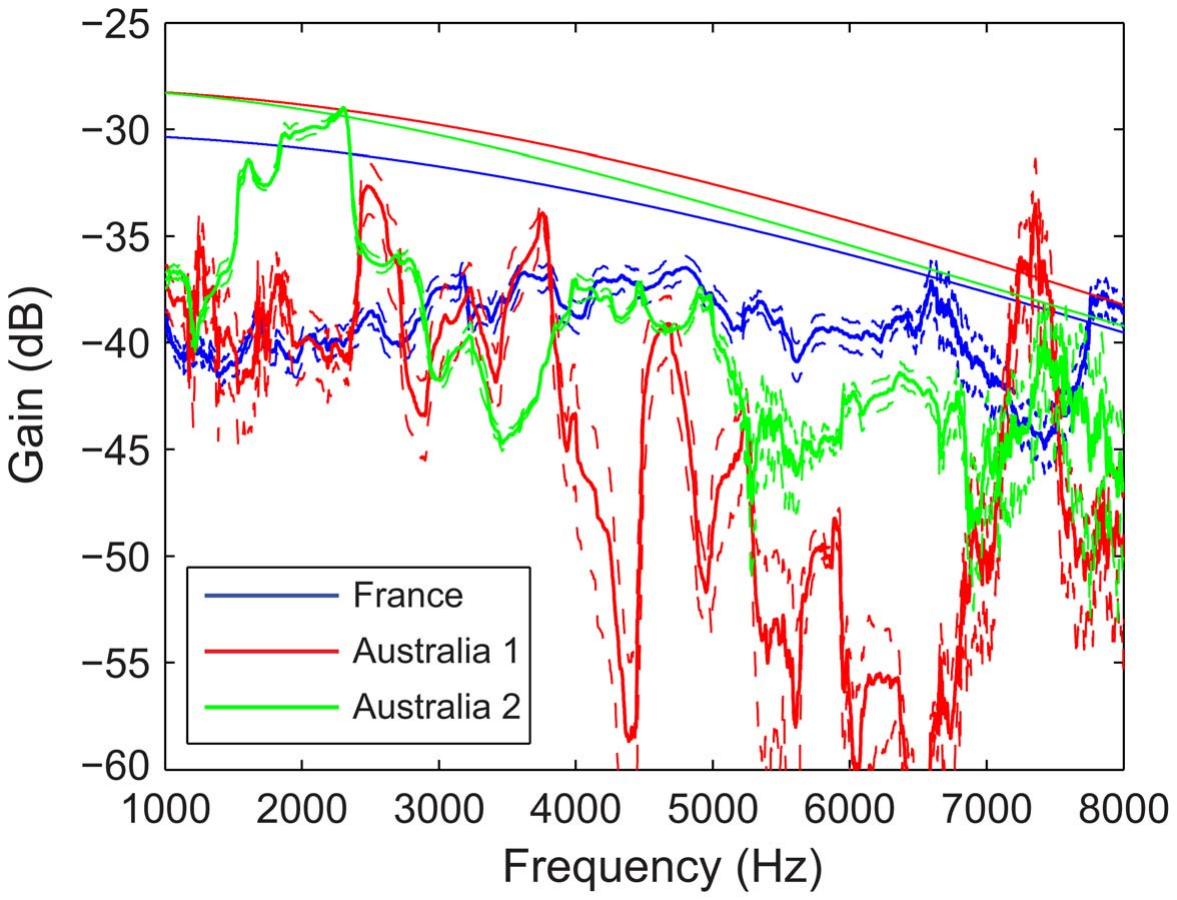
Comparison of transfer functions in France and in Australia. The transfer functions were calculated between the recordings obtained at 2 = 30°C, 15% relative humidity and 1 atm. The Australia site 2 is next to zebra finch nesting sites and includes sparse vegetation and the recordings were performed on a windless evening. The attenuation curve was obtained using T = 25°C, 15% relative humidity and 1 atm. The recordings in France are in an open field and were obtained during the day in low wind conditions. The attenuation curve was obtained using T = 11°C, 50% relative humidity and 1 atm.

To what extent are these measurements affected by the environment? On Fig. 5, we show the frequency transfer function between 2 and 64 m for calls recorded in France and between 2 and 50 m for the sounds recorded in Australia. The transfer function between a call recorded at 2 m and the same call recorded at 64 m shows the attenuation of the signal as a function of frequency, taking into account all environmental effects: terrain, vegetation, temperature, ground effects, etc. The frequency transfer function in France is relatively flat between 1 and 7 kHz, at 10 to 5 dB below the attenuation expected from spherical spreading and atmospheric absorption (thin solid blue line on Fig. 5). Thus, only 5 dB to 10 dB attenuation can be attributed to other factors such as wind, temperature gradients, ground effect, terrain and vegetation. The transfer functions obtained from the recordings performed at site 2 in Australia (green lines) have more structure, with variations alternating between 0 and 15 dB below the value expected from spherical spreading and atmospheric absorption. We attribute most of the variations to the sparse vegetation that was found at that location. The recordings at site 1 (red lines) were performed in a more open region and the corresponding transfer function shows different fluctuations at low frequencies and a greater attenuation at frequencies above 4 kHz. These measurements show, as it is well known, that the attenuation of sound in a natural environment deviates from theoretical values because of specific terrain, landscape and other effects such as reflections from the ground and temperature gradients. However, the deviations from spreading and atmospheric absorption observed in Australia are relatively small and are likely to be idiosyncratic to very local conditions (mostly vegetation). For these reasons, the frequency transfer function of the terrain in France can be considered to be a relatively good surrogate of an “average” of open terrains in Australia that wild zebra finches encounter. The rest of the results will focus on the analyses performed with the recordings obtained in France.

### The modulation power spectrum of degraded calls

**Figure 6:**
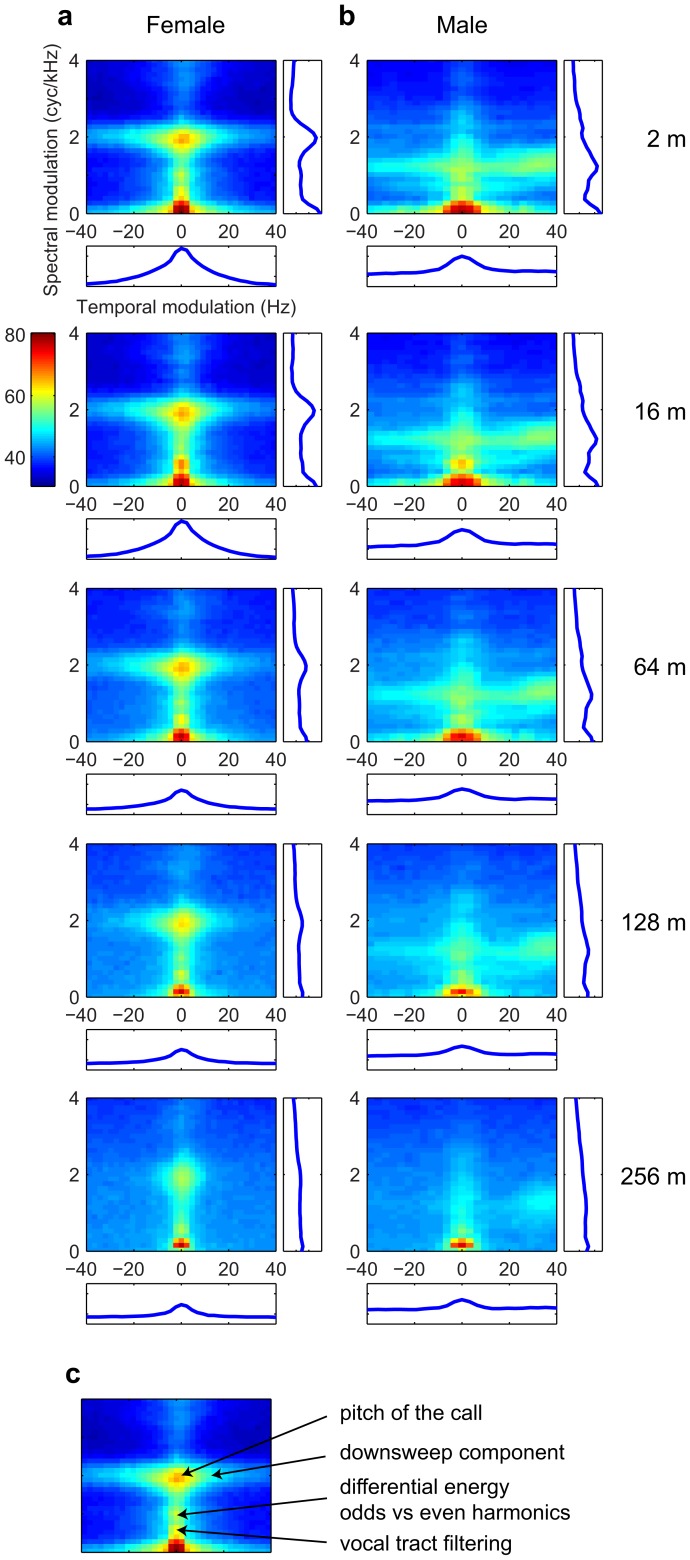
Modulation power spectra (MPS) of female (a) and male (b) calls for each propagation distance. Mean MPSs are shown for each propagation distance used in this study (from 2 m to 256 m). Projections on the spectral modulation axis (in cycles per kHz) and the temporal modulation axis (in Hz) are shown beside and below each MPS respectively. The major observable features are shown and labeled on one example shown in panel (c).

While the spectrograms and frequency power spectra of the propagated calls illustrate the changes in SNR as function of frequency and distance, they fail to quantify how this attenuation affects potentially informative spectral and temporal structures in the signal. To quantify these structural changes, we calculated the modulation power spectrum (MPS) of the calls, which is the 2-D power spectrum of the joint spectro-temporal envelope of the sound [27] (see Methods). On Fig. 6 we show the average MPS of all calls at each distance, for each sex. Temporal modulations are shown on the x-axis and characterize the power in the temporal amplitude envelope as a function of temporal frequencies (in Hz); signals that contain fast changes in their envelope (such as a quick onset or a fast chirp) are characterized by power at the higher temporal modulation frequencies. The spectral modulations are shown on the y-axis and characterize the power in the spectral envelope in terms of spectral frequencies (in cycles per kHz); signals with clearly defined harmonics show power at the spectral modulations corresponding to the inverse of the fundamental frequency (higher for lower pitch). Noisy broadband sounds show broad power at low spectral modulations. Any point away from these axes represents joint spectro-temporal modulations such as upsweeps (in the area with negative temporal modulation) and downsweeps (in the area with positive temporal modulation) [27].

For the females (Fig. 6a), the MPS at short distances (2 m and 16 m) show a large area of energy around 2 cyc/kHz and very low temporal modulations (centered at 0 Hz), corresponding to the salient and relatively steady pitch of the harmonic stack in the call; the spectral modulation value of 2 cyc/kHz corresponds to a fundamental frequency of around 500 Hz, consistent with the mean fundamental frequency of the female distance calls used in our dataset. The spread of energy at 2 cyc/kHz along the horizontal dimension represents the up- and down-sweeps at the beginning and end of the calls. The smaller peak of energy around 1 cyc/kHz is characteristic of differential energy in the odd vs. even harmonics (1 cyc/kHz corresponds to a frequency of 1 kHz, which characterizes a boost in energy every second harmonic) and energy below 0.5 cyc/kHz corresponds to the spectral envelope produced by the filtering of the upper vocal tract (the equivalent of the formants; Fig. 6c, [30]).

Fig. 6a clearly shows that for the females, the joint spectro-temporal modulations corresponding to the up- and down-sweeps are progressively lost with propagation distance, while the energy in the pitch area remains visible at 256 m, albeit less prominently. These degradations are also evident on the spectrogram for the example call in Fig. 3: the faster modulation changes found at the beginning and end of the female call fall more rapidly below the noise ceiling than the louder and less dynamic central section of the call. The relative decrease of energy in the pitch area is a consequence of the loss of the lower and higher harmonics as well as a decrease in the sharpness of the remaining harmonics in the 2–4 kHz region.

For the males (Fig. 6b), information corresponding to the pitch of their harmonic stack is also clearly visible, between 1 and 2 cyc/kHz, which is consistent with the higher fundamental frequency of their calls around 700 Hz. Similarly to what is also observed in females but to a much greater extent, the energy corresponding to the up- and down-sweeps is visible. In particular, a large bulge of energy is found on the right side of the y-axis corresponding to the fast down-sweeps that characterize the trailing end of the male call. While the information about up-sweeps is progressively lost (as was the case for the females), these high frequency down-sweeps, with a modulation rate of about 25 to 40 Hz, remain clearly visible up to 256 m. These modulations, characteristic of the male call, preserve the sexual dimorphism of the calls at all distances. Finally, for both males and females, the energy visible along the x-axis (corresponding to the overall temporal envelope) is progressively reduced to lower modulation frequencies with distance; thus, sound propagation also degrades the faster dynamics in the temporal envelope.

In summary, the sharp spectral features of the harmonic stack in the female call are relatively well preserved at long distances, while information about the more dynamic parts of the calls (i.e., the spectro-temporal modulations at the beginning and end of the signal) is more rapidly lost with distance. For male calls, both the information in the harmonic structure of the harmonic stack as well as the high frequency downsweep component are relatively well preserved, which maintains the sexual dimorphism of these propagated distance calls.

### Effect of propagation on individual vocal signatures

The nature of the individual information in the distance call and the way this information degrades with distance were assessed and quantified using a cross-validated linear discriminant function analysis (see Methods and Fig. 2). These discriminant analyses were performed under three scenarios. First, we analyzed the data separately for each distance, in order to obtain ceiling values for discrimination through quantifying correct classification in validating datasets also obtained from the corresponding distance. This analysis simulated a scenario where the bird could use the actual distance of the sound source as independent information to discriminate individuals. We then conducted two additional analyses that allowed us to determine putative discrimination abilities in simulated scenarios where the bird would not have access to independent information on the actual distance of the sound source. In one scenario, we considered the hypothesis that building up on their experience of recognizing a number of individuals with which they had many interactions, mostly at close range, birds would be able to recognize these same individuals at longer distances. We tested a model where the bird uses the acoustical features most useful for identity discrimination at short distance for all of its discriminations: we used the calls recorded at 2 m as a reference for analyzing calls recorded at all other distances. In the other scenario, we tested a model where the bird also uses the same acoustical features at all distances but where these features are found by taking all propagation distances into account: we used all calls recorded irrespective of distance (and ignoring this information) for analyzing individual discrimination.

#### 1. Discriminant analysis for each distance separately

**Figure 7:**
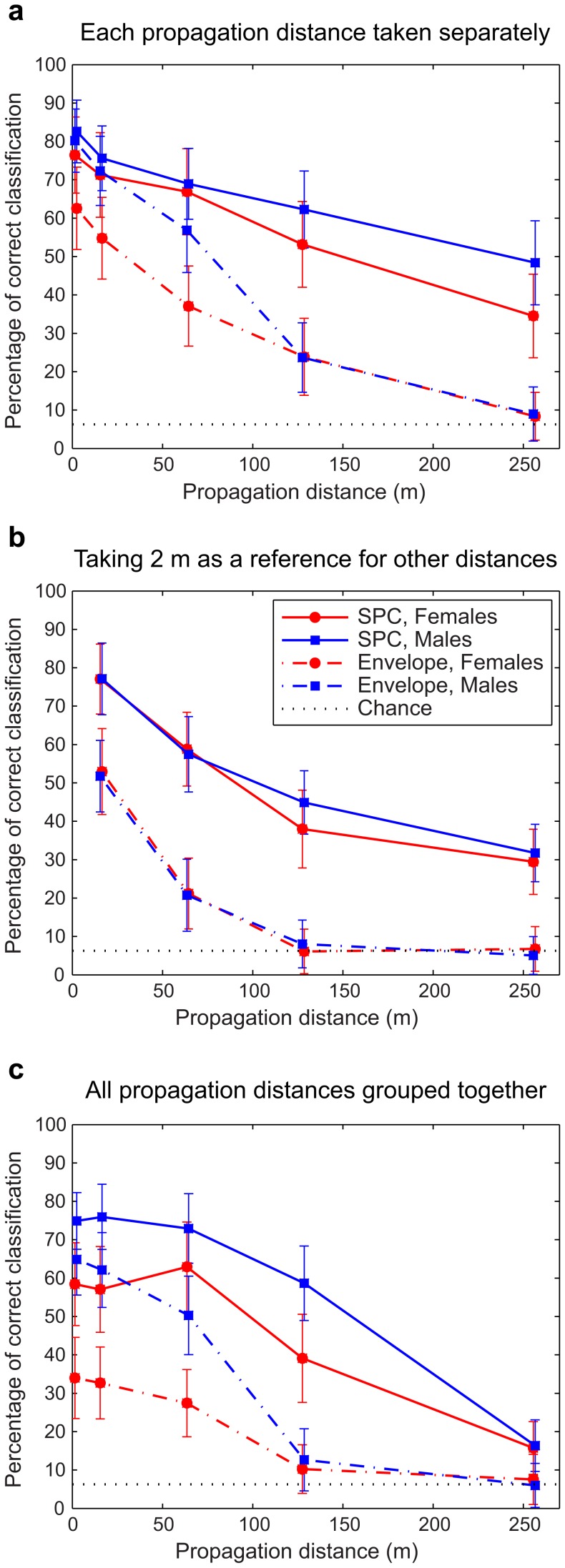
Mean percentages of correct classification obtained for each propagation distance and both sexes. (a) Taking the data for each propagation distance separately, (b) taking the 2 m data as a reference for other distances, and (c) taking all propagation distances into account. SPC parameters are represented as solid lines and envelope parameters as dash-dot lines. Standard deviations are indicated. The chance level, corresponding to 6.25% of correct classification, is shown as a horizontal dotted line.

On Fig. 7a, we show the percentages of correct classification as a function of distance, for both sexes and both sets of parameters (envelope and SPC) when the discriminant analysis is performed for each distance separately. Using the envelope parameters, the percentages of correct classification at 2 m average 80.2% for male calls (n = 16 individuals), and a noticeably lower value of 62.6% for female calls (n = 16 individuals). These values increase when the SPC representation is used, averaging 82.6% for the males and 76.5% for the females (Fig. 7a – values at 2 m). These classification values remain above chance for most other distances. However, when the envelopes parameters are used to describe the calls, the percentage of correct classification obtained at 256 m is close to chance level for both male and female calls. Statistical analysis shows that there is a significant effect of distance (ANCOVA with the percentage of correct classification as the dependent variable, sex and type of parameter as factors and distance as a covariate; *p*<10^−6^). Discrimination is thus more and more difficult with increasing distances (with an average decrease of 1.94% points every 10 m). There is also an effect of sex on the percentage of correct classification, the discrimination of female calls being on average 9 percentage points below those of males (*p* = 0.02). Finally, there is a significant effect of the type of parameter, with the envelope parameters yielding worse performance by 21 percentage points (*p*<10^−4^). When interactions are included in this linear model, one also finds that the percentage of correct classification decreases more rapidly with distance for the envelope parameters than for the SPC parameters (*p* = 0.009). Further analysis taking into account each sex separately shows that this difference between the two slopes is due to the males only (F = 10.24, *p* = 0.0186): the performance of the discrimination deteriorates at a faster rate for the first distances (up to 128 m) with the envelopes parameters compared to SPC parameters.

In summary, this analysis shows that the males' individual signature is stronger than the females' and that for both sexes, using the SPC parameters give significantly better results in terms of discriminability than using the envelope parameters, especially at longer propagation distances.

#### 2. Discriminant analysis taking 2 m as a reference: maximizing peak discrimination

Here, discriminability between individuals decreases more rapidly with distance than for the previous analysis, the slope of the curves being less linear (Fig. 7b). Specifically, discriminability decreases on a steeper curve at medium to long range for the SPC parameters but still remains well above chance level at 256 m for both sexes. For the envelope parameters, the percentages of correct classification at short range are well below those obtained in the previous analysis, and by 128 m discriminability is equivalent to chance for both sexes. Indeed, the ANCOVA analysis using the percentage of correct classification as the dependent measure, the type of parameter used in the DFA and the sex as factors and distance as the covariate showed a significant main effect for the type of parameters (*p*<10^−4^) with an estimate decrease of 30 percentage points for the envelope parameters relative to the SPC parameters, but no significant interaction between the type of parameter and sex. In this case, we failed to find a significant main effect for sex.

In summary, while the use of SPC parameters always gives better results than envelope parameters, taking 2 m as a reference is less efficient than the analysis where each distance is taken separately, the percentages of correct classification decreasing more rapidly. The enhanced discriminability of male vocal signatures observed in the latter analysis disappears when 2 m is taken as a reference.

#### 3. Discriminant analysis of all propagation distances grouped together: maximizing average discrimination

The percentages of correct classification shown on Fig. 7c quantify the discriminability obtained using a single set of DFs based on data from all distances. As it is the case when the DFA is performed for each propagation distance separately, the ANCOVA shows that there is a main effect of distance, sex and parameter type. Moreover the estimated values are very similar (compare 7c with 7a): the increase in distance results in 1.9 percentage points loss per 10 m (*p*<10^−6^), discrimination of male calls is superior than female calls by 14.9 percentage points (*p* = 0.002) and envelope parameters give percentages of correct classification that are on average 22.4 points worse than the discrimination obtained with the SPC parameters (*p*<10^−4^). Thus, this strategy results in relatively small penalties relative to the strategy that involves using the optimal DFs at each distance, but only when the SPC parameters are used (compare with 7a): the difference in the percentages of correct classification was within our estimate of the standard error for all distances except 256 m. For the envelope parameters, the decrease in performance for the males call was also small and within the estimated standard error, while for the females we notice substantially lower discrimination rates at short distances (2 m and 16 m).

These results show that as long as the SPC parameters are used, a single distance-independent template can give very good results in terms of discriminability, with a noticeable drop occurring only at 256 m. Thus, although different acoustical features (as will be described in more detail below) could be used to optimize discrimination for different propagation distances, we also found a strategy using a single and robust set of acoustical features that yields good discrimination at all distances.

#### 4. Acoustical parameters used for identity discrimination

**Figure 8:**
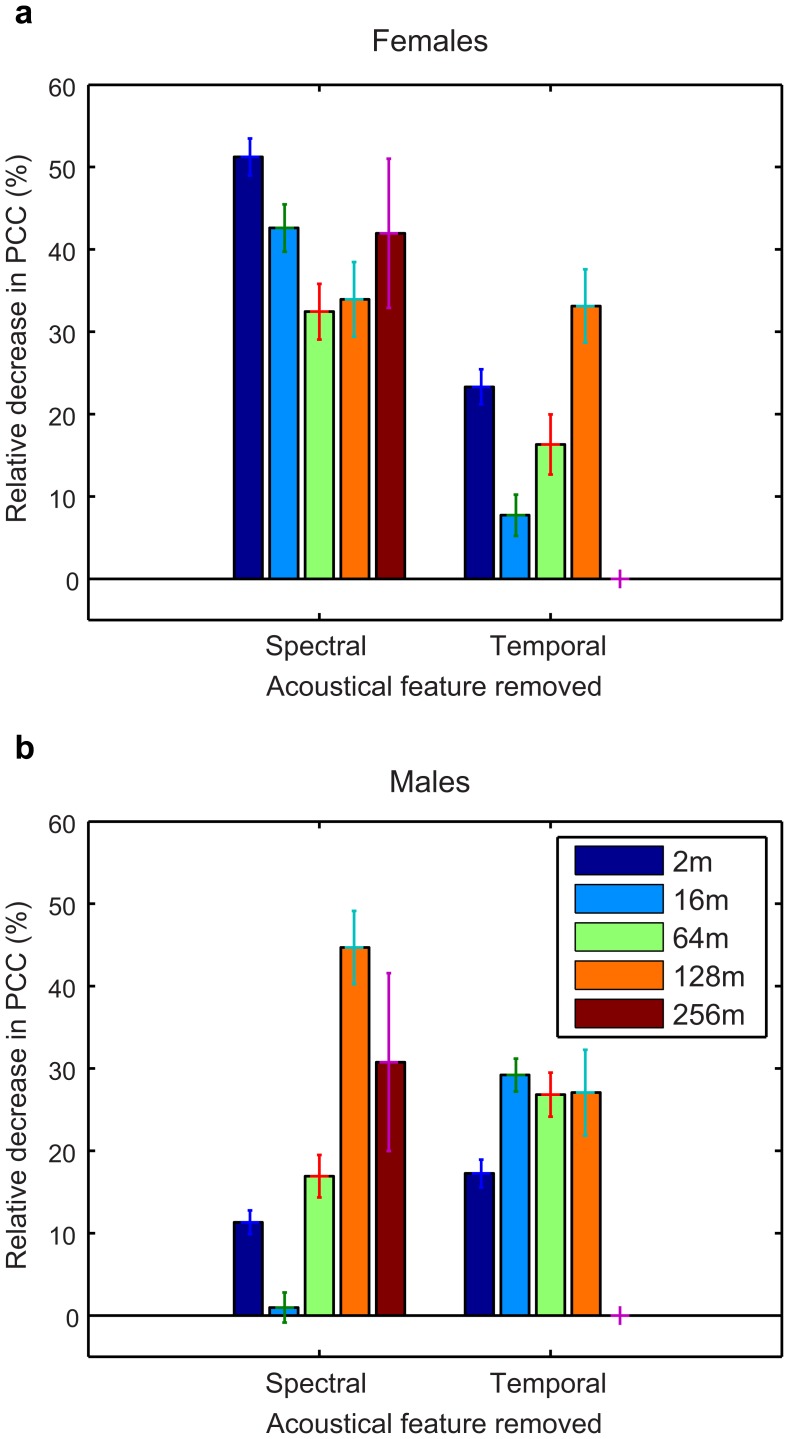
Comparison between the importance of spectral and temporal features of the envelope parameters. Calculations are shown separately for females (a) and males (b). Histograms represent the relative decrease as a percentage in the probability of correct classification (PCC) observed when removing the spectral (left) or temporal (right) parameters from the DFA. Error bars show two-standard errors estimated by bootstrapping. For each distance, except for 128 m for females, the changes observed by removing spectral information vs. the temporal information are significantly different (all *p* values <10^−5^ using a two-sided t-test).

As can be seen on Fig. 8, the spectral envelope carries more information than the temporal envelope for females at 2, 16 and 64 m while the opposite is true for males. At 128 m, spectral and temporal envelope features are equally informative in females whereas spectral envelope features start carrying more information than temporal features for males. At 256 m, temporal features carry no more information for both sexes. Additionally, the relative importance of temporal or spectral parameters does not obey monotonic functions of distance. For example, discriminability of female calls at 2 m is higher than at 16 m but is also more relatively affected by the elimination of temporal features; as a result, the overall discriminability of female calls based only on spectral features becomes very similar between 2 and 16 m. In brief, informative features follow a clear sexually dimorphic pattern with not only differences in the relative importance of spectral vs. temporal features between the sexes but also opposite trends as a function of increasing propagation distance. This analysis also sheds light on the redundancy in the information in the temporal vs. spectral envelope. If the information was completely independent, the spectral and temporal bars shown on Fig. 8 would add up to 100%. The data show that there is always some redundancy in the spectral and temporal envelope information but 1) that this redundancy is higher in males that in females and 2) that it decreases for males while it increases slightly for females as propagation distance increases.

**Figure 9:**
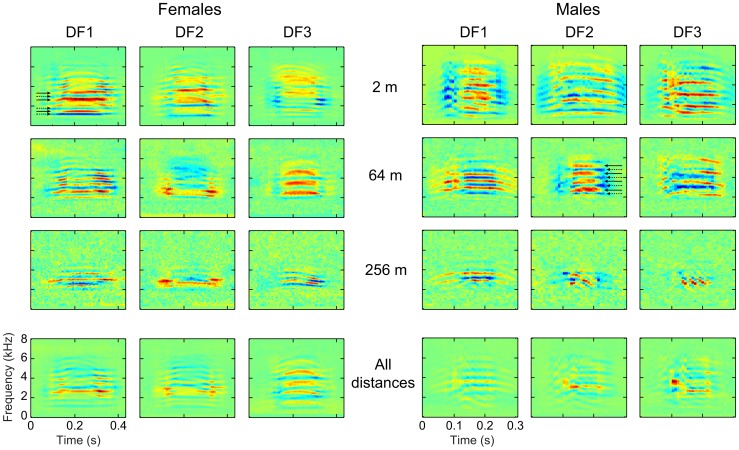
Representation of the discriminant functions (DFs) projected into the spectrographic space for the SPC parameters. The first 3 DFs, obtained from the SPC parameters, are shown for females (left) and males (right). Each row indicates the type of dataset used to perform the DFA: each distance taken separately for 2 m, 64 m and 256 m (first 3 rows) and all distances taken into account (bottom row). As examples, on DF1 at 2 m for females and DF2 at 64 m for males, full arrows show positive frequency bands (red) and dashed arrows show negative bands (blue). This representation enables a description of the most important features in the spectrogram that can be used to discriminate between individuals at various distances.

A more explicit description of the effect of propagation on the informative features can be obtained with SPC parameters, by analyzing the evolution with distance of the DFs represented in the spectrographic space (Fig. 9). One striking result of that visualization is that both the range of the frequency spectrum and the duration in time where intensity is available shrinks as distance increases. While a large range of the frequency spectrum (between approximately 1 and 6 kHz) can be used to extract the individual signature at 2 m, the frequency band of the signal then shrinks as a function of distance and becomes restricted to a narrow range between 2 and 4 kHz at 256 m. The decrease in the temporal range is most evident for DF3 and more pronounced for the male calls: while the temporal range of the male DF3 for 64 m is above 200 ms it drops to below 100 ms at 256 m.

The DFs in Fig. 9 also show that fine spectral cues (the narrower alternating blue and red bands in the frequency bands) appear to be useful at all distances, and for both sexes. As explained above, this spectral structure is crucial for the pitch percept of the call. Thus, even though the bandwidth which can be used to extract this pitch information is reduced, this analysis shows that pitch information remains robust, a testament to its redundant character in broadband harmonic stacks. One feature that strikingly sets sexes apart at long range is the selectivity for downward sweeps that appears for males in DF2 and DF4 at 128 m (not shown on the figure) and in DF2 and DF3 at 256 m. These sweeping sound features are clearly not present for female calls, and are also less crucial for discriminating male calls when the entire frequency range of the signal is available. The detection of these information bearing joint spectro-temporal features is only feasible when the complete spectrographic representation of the sound (SPC parameters) is used in the discriminant function analysis.

Finally, we can also compare the DFs obtained at each distance to the single set of DFs that could be used to discriminate the caller identity irrespective of distance (bottom row of Fig. 9). The DFs obtained with the analysis taking all distances into account show that fine spectral cues are indeed important for discrimination, for both sexes; temporal patterns are also clearly visible for females, and to a lesser extent for males. Finally, one can note that while the “all-distances” DFs sample a relatively large frequency bandwidth, as seen in the DFs optimized for short distances, the weighting in these DFs optimizes the same narrower frequency range as in the DFs optimized for long distances (between 2 and 4 kHz). In this respect, the “all-distances” DFs illustrate the template of information within the spectrographic space that could be taken into account in order to achieve optimal discrimination at all distances.

In summary, informative features about identity are coded redundantly in distance calls and are stronger in male than in female calls. While the frequency and temporal ranges of the informative features in the calls decrease with distance, fine spectral cues are useful for all distances. Finally, information about downward sweeps is important to discriminate between male calls at long distance, but not crucial at shorter distances, further exemplifying the information redundancy and sexual dimorphism.

### Comparison between frequency power spectrum of calls and frequency gain of discriminant functions

**Figure 10:**
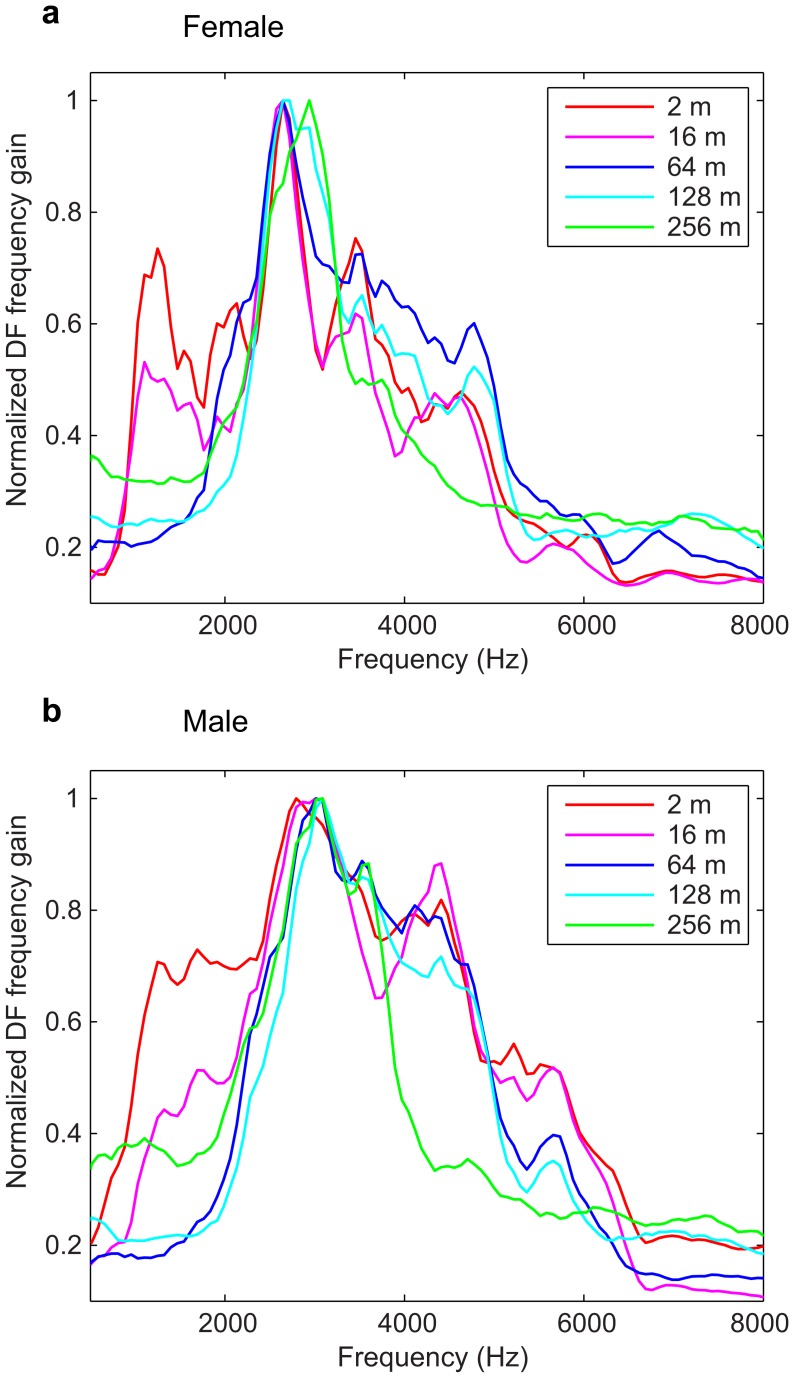
Relative importance of the discriminant functions (DFs) as a function of frequency. Calculations are shown for females (a) and males (b), for every propagation distance. These *importance* functions were obtained by calculating the normalized mean of the absolute values of the first 4 DFs for each frequency window. This analysis shows the frequencies that are the most useful to discriminate between individuals at each distance.

In Fig. 4, we have shown the effect of propagation on the frequency power spectrum of the zebra finch distance call. Here we compare the frequency dependent SNR shown in those power spectra with the frequency gain of the discriminant functions: we calculated the normalized means of the absolute values of the DF for each frequency window (taking into account the first 4 DFs, weighted with their corresponding eigenvalues) and plotted this relative importance as a function of frequency. The results of that calculation are shown on Fig. 10. For close distances (2 m and 16 m), the “critical band-pass” in the signal that is of importance for individual discrimination is broad, ranging from approximately 700 Hz to 6 kHz. At further distances (64 m and 128 m) this critical band-pass is reduced mostly in the lower frequencies, ranging from approximately 2 to 6 kHz, and at 256 m the interval is reduced again, mostly in the higher frequencies this time, ranging from approximately 2 to 4 kHz. Thus, both the peaks of these DF frequency gain curves and their bandwidth match the shape of the FS of the calls with a peak approximately at 2.7 kHz for the females and 3 kHz for the males and a decrease in bandwidth as the propagation distance increases. However, one can also observe that the bandwidth of the DF frequency gain curves does not decrease as linearly as the FS of the calls: the gain remains high until the SNR drops below a certain level. This effect is clear for example for the information between 500 Hz and 2 kHz that is much higher in the DF frequency gain curves than what one would predict from the frequency spectra; similar effect is shown at higher frequencies (between 5 and 6 kHz). By examination of the DFs, one can see that this lower frequency shoulder includes spectral information on the pitch of the call (corresponding to the 2^nd^ harmonic) that is particularly well resolved at these frequencies. The higher frequency shoulder appears to have both fine and coarse spectral information (pitch and formants).

In summary, this analysis shows that the relationship between SNR and the gain of filters that can extract specific information in the signal is not proportional in the way that it would be for a simple detection task (and given, for example, by the Wiener filter for Gaussian signals). Instead, the frequency gain curve for a discrimination task must also take into account the frequency dependent weight of the information content.

## Discussion

Our analysis shows that the individual signature encoded in the distance calls of zebra finches is remarkably resistant to propagation-induced sound degradation: after more than a hundred meters of propagation, distance calls still allow for a good discrimination between individuals, and even after propagating over 256 m it remains well above chance when using acoustical features optimized for that distance. In addition, we have confirmed that information about the individual identity is carried redundantly, using multiple acoustical features [9], [21]. Moreover, the features providing the highest discrimination at long distances are not the same as those that provide the highest information at short distances. On one hand, the redundancy could therefore be used to optimize information transmission at different distances. On the other hand, we also found that a single set of acoustical features could be used to maximize information about identity irrespective of distance (except for the furthest, 256 m). Finally, whereas discriminability for both sexes is identical at long range using the parameters of the envelopes (extracted from temporal or spectral domains), male calls show a stronger individual signature at long distance compared to female calls when using the SPC parameters (extracted from the whole spectrogram of the calls). This sexual dimorphism is not due to higher signal power in the male call but to structural differences in the distance calls of each sex. These structural differences do not seem to affect the discriminability between individuals for either sex at close and medium range, but may provide an advantage to male signature over longer distances.

The results of the discriminant analysis confirm and complete our knowledge on the sexual dimorphism in the individual signatures of the zebra finch distance call. In males, the faster and more predominant downsweep component appears to become critical for discriminability at long distances. Interestingly, while the DFs for the females are comparable in structure to spectrograms of female calls, the DFs corresponding to the males are very different from the spectrographic representation of an average male call, the downsweep component in particular being absent for short and medium distances and only appearing at 128 m and 256 m. These fine spectro-temporal modulations appear therefore less critical for discrimination at short and medium distances. Information about frequency modulation is therefore not necessary at close range. The observation that the information embedded in the parameters from either the spectral or the temporal envelope is not independent (Fig. 8) further confirms this conclusion. An acoustic analysis using parameters describing the modulation of the fundamental frequency would indeed find that the downsweep component of male calls is highly individualized [9]; what our study highlights however, is that the same level of individualization can be found at close range in male calls using parameters describing only its energy spectrum and its temporal variation in amplitude. Concerning female calls, Vignal *et al.* [8] found that a variety of spectral parameters describing the frequency modulation of the fundamental frequency were highly individualized. As shown on Fig. 7a, the higher discriminability in the female calls found for the SPC parameters compared to the envelope parameters indeed shows that information about frequency modulation is important for discrimination between females, even at short range, which is in accordance with previous work.

The frequency spectrum (FS) of our propagated calls show that the band-pass of the signals is progressively reduced with distance. The transfer function calculation shows that the frequency dependent attenuation is relatively flat in the region of interest and that the attenuation can be explained within 5 to 10 dB by spherical spreading and atmospheric absorption. Therefore, the resulting band-pass quality of the FS (Fig. 4) and the matched frequency gain of the DFs (Fig. 10) can be simply explained by the FS of the calls that is also band-pass limited, the FS of the noise that decreases with frequency and a relatively flat attenuation (slowly increasing with frequency as shown in Fig. 3) due to propagation [31]. In an environment where the spectrum of the background noise would have a significantly different shape or where the terrain and vegetation would result in a different transfer function profile, the informative frequency channel could be different. In addition, other effects such as reflection from the ground and inhomogeneous atmospheres (e.g. from temperature gradients) could also have an effect. At our source-receiver elevations of 1.3 m these effects appear to be negligible [5], [32] but since zebra finches also spend time on the ground and at higher elevations, propagation studies in such conditions could reveal additional important factors in natural propagation and other specificities of informative features in distance calls. However, as described above, our analysis using recordings performed in an environment where the noise spectrum is typical of a low wind day in an open field with relatively little vegetation can be considered to be typical of the scenario encountered by the zebra finch in its native environment emitting calls and perceiving calls in bushes and small trees.

Interestingly, a study using synthesized calls of zebra finches have shown that only a small number of harmonics in the distance call is necessary to elicit a behavioral response from the other sex [33]; for both males and females, this minimum bandwidth (3200 to 3500 Hz) fits inside the bandwidth containing individual information that we found at 256 m (2000 to 4000 Hz). The match between calls features and environmental characteristics has been the subject of extensive research about the influence of the acoustic properties of the environment on the selection of acoustic signals. Morton [34] suggested that the design of acoustic signals should maximize their transmission distance with regards to the characteristics of the habitat. For best resistance to degradation in an open environment, an ideal call should be short and have a high frequency modulation rate [35]. The distance calls of male zebra finches typically present these qualities, and our analysis shows that their individual signature remains stronger at long distance compared to the female calls. In this light, the fact that the females' calls are not more frequency modulated raises the question of partners' recognition. It is possible that partners rely more on the male's call, whose individual signature is more resistant to degradation, to locate each other. Indeed, Zann observed that males do call more frequently than females [19]. To investigate this hypothesis one could study the dynamics of movements when the mates reunite after being separated visually but not acoustically, and test if the females move more towards the males than the opposite, depending on the distance between mates or the level of degradation of the calls. Besides, it has been demonstrated that male zebra finches increase their vocal amplitude with increasing distance to addressed females [36]: this ability may further increase the active space of the signature embedded in male calls, and females could also increase the amplitude of their calls to compensate for their weaker individual signature at long distance.

Only playback experiments will ultimately allow testing if male calls ensure better partner's recognition than female calls at very long distances. However, adaptation to the habitat characteristics is not the only criteria in the selection of vocal signals [37]–[39]. As the distance call is a learned vocalization in males (contrary to the females), its sex-specific characteristics may be a by-product of song learning, rather than the result of a strong selective shaping for long distance propagation. Influences other than habitat adaptation, such as sexual selection or learning processes, may have mainly induced the acoustic differences found in the calls of both sexes.

At the other end of the transmission chain, we can now consider the perspective of a bird decoding the individual identity in a propagated signal. As described in the results, one can imagine that the decoder could use a distance dependent set of acoustical features to determine the call identity. This strategy would maximize discriminability of identity but perhaps with the cost of the additional cognitive processing required to extract a large set of high-level acoustical features. Alternatively, we have shown that a single set of features obtained to maximize the average discrimination over a range of behaviorally relevant distances performs exceptionally well. Birds may therefore adopt a strategy where only one set of acoustical features is used for all distances. Behavioral experiments, assessing the psycho-auditory abilities of birds in discriminating between various degraded acoustic signals, or using synthetic sounds where particular acoustical features are systematically manipulated, could be designed to answer these questions.

## Supporting Information

Audio File S1 **Examples of propagated distance calls of males and females.** Two examples for each sex are broadcasted at 2 m, 64 m and 256 m.
(WAV)

## References

[1] Forrest TG (1994) From sender to receiver: propagation and environmental effects on acoustic signals. Integr Comp Biol 34: 644–654 10.1093/icb/34.6.644

[2] Wiley RH, Richards DG (1982) Adaptations for acoustic communication in birds: sound transmission and signal detection. In: Kroodsma DE, Miller EH, editors.Acoustic Communication in Birds.New York: Academic Press, Vol. 1 . pp. 131–181.

[3] Brenowitz EA (1982) The active space of red-winged blackbird song. J Comp Physiol A: Neuroethology, Sensory, Neural, and Behavioral Physiology 147: 511–522.

[4] Lohr B, Wright TF, Dooling RJ (2003) Detection and discrimination of natural calls in masking noise by birds: estimating the active space of a signal. Anim Behav 65: 763–777 10.1006/anbe.2003.2093

[5] Marten K, Marler P (1977) Sound transmission and its significance for animal vocalization. Behav Ecol Sociobiol 2: 271–290 10.1007/BF00299740

[6] Charlton BD, Reby D, Ellis WAH, Brumm J, Fitch WT (2012) Estimating the Active Space of Male Koala Bellows: Propagation of Cues to Size and Identity in a Eucalyptus Forest. PLOS ONE 7: e45420 10.1371/journal.pone.0045420 23028996 PMC3447879

[7] Robertson BC (1996) Vocal mate recognition in a monogamous, flock-forming bird, the silvereye, *Zosterops lateralis* . Anim Behav 51: 303–311 10.1006/anbe.1996.0030

[8] Vignal C, Mathevon N, Mottin S (2004) Audience drives male songbird response to partner's voice. Nature 430: 448–451 10.1038/nature02645 15269767

[9] Vignal C, Mathevon N, Mottin S (2008) Mate recognition by female zebra finch: analysis of individuality in male call and first investigations on female decoding process. Behav Proc 77: 191–198 10.1016/j.beproc.2007.09.003 17980974

[10] Lovell SF (2004) Neighbor-stranger discrimination by song in a suboscine bird, the alder flycatcher, *Empidonax alnorum* . Behav Ecol 15: 799–804 10.1093/beheco/arh082

[11] Naguib M, Todt D (1998) Recognition of neighbors' song in a species with large and complex song repertoires: the Thrush Nightingale. J Av Biol 29: 155–160.

[12] Jouventin P, Aubin T (2002) Acoustic systems are adapted to breeding ecologies: individual recognition in nesting penguins. Anim Behav 64: 747–757 10.1006/anbe.2002.4002

[13] Jouventin P, Aubin T, Lengagne T (1999) Finding a parent in a king penguin colony: the acoustic system of individual recognition. Anim Behav 57: 1175–1183 10.1006/anbe.1999.1086 10373249

[14] Aubin T, Mathevon N, Silva ML, Vielliard JME, Sèbe F (2004) How a simple and stereotyped acoustic signal transmits individual information: the song of the white-browed warbler *Basileuterus leucoblepharus* . Anais da Academia brasileira de Ciencias 76: 335–344. 15258647 10.1590/s0001-37652004000200022

[15] Mathevon N, Aubin T, Vielliard J, da Silva M-L, Sèbe F, et al (2008) Singing in the rain forest: how a tropical bird song transfers information. PLOS ONE 3: e1580 10.1371/journal.pone.0001580 18270571 PMC2216701

[16] Rek P, Osiejuk TS (2011) No male identity information loss during call propagation through dense vegetation: the case of the corncrake. Behav Proc 86: 323–328. 10.1016/j.beproc.2011.01.011 21295119

[17] Butterfield PA (1970) The pair bond in the zebra finch. Social behaviour in birds and mammals: Essays on the social ethology of animals and man. London: J. H. Crook.pp. 249–278.

[18] Zann RA (1996) The zebra finch: a synthesis of field and laboratory studies. Oxford: Oxford University Press.

[19] Zann R (1984) Structural variation in the zebra finch distance call. Zeit Tierpsychol 66: 328–345 10.1111/j.1439-0310.1984.tb01372.x

[20] Vicario DS, Naqvi NH, Raksin JN (2001) Sex differences in discrimination of vocal communication signals in a songbird. Anim Behav 61: 805–817 10.1006/anbe.2000.1651

[21] Forstmeier W, Burger C, Temnow K, Derégnaucourt S (2009) The genetic basis of zebra finch vocalizations. Evolution 63: 2114–2130 10.1111/j.1558-5646.2009.00688.x 19453380

[22] Jacot A, Reers H, Forstmeier W (2010) Individual recognition and potential recognition errors in parent–offspring communication. Behav Ecol Sociobiol 64: 1515–1525 10.1007/s00265-010-0965-5

[23] Mulard H, Vignal C, Pelletier L, Blanc A, Mathevon N (2010) From preferential response to parental calls to sex-specific response to conspecific calls in juvenile zebra finches. Anim Behav 80: 189–195 10.1016/j.anbehav.2010.04.011

[24] Griffith SC, Pryke SR, Mariette M (2008) Use of nest-boxes by the Zebra Finch (*Taeniopygia guttata*): implications for reproductive success and research. Emu 108: 311–319 10.1071/MU08033

[25] Perez EC, Elie JE, Soulage CO, Soula HA, Mathevon N, et al (2012) The acoustic expression of stress in a songbird: Does corticosterone drive isolation-induced modifications of zebra finch calls? Hormones Behav 61: 573–581 10.1016/j.yhbeh.2012.02.004 22387308

[26] Thomson D (2007) Jackknifing Multitaper Spectrum Estimates. IEEE Signal Proc Mag 24: 20–30 10.1109/MSP.2007.4286561

[27] Singh NC, Theunissen FE (2003) Modulation spectra of natural sounds and ethological theories of auditory processing. J Acoust Soc Am 114: 3394–3411 10.1121/1.1624067 14714819

[28] Okanoya K, Dooling RJ (1987) Hearing in passerine and psittacine birds: a comparative study of absolute and masked auditory thresholds. J Comp Psychol 101: 7–15 10.1037/0735-7036.101.1.7 3568610

[29] Mathevon N, Koralek A, Weldele M, Glickman SE, Theunissen FEE (2010) What the hyena's laugh tells: Sex, age, dominance and individual signature in the giggling call of *Crocuta crocuta* . BMC Ecol 10: 9. 20353550 10.1186/1472-6785-10-9 PMC2859383

[30] Elliott TM, Theunissen FE (2009) The modulation transfer function for speech intelligibility. PLoS comput biol 5: e1000302. 19266016 10.1371/journal.pcbi.1000302 PMC2639724

[31] Wiley RH (1991) Associations of song properties with habitats for territorial oscine birds of Eastern North America. Am Nat 138: 973–993.

[32] Cosens SE, Falls JB (1984) A comparison of sound propagation and song frequency in temperate marsh and grassland habitats. Behav Ecol Sociobiol 15: 161–170 10.1007/BF00292970

[33] Vignal C, Mathevon N (2011) Effect of acoustic cue modifications on evoked vocal response to calls in zebra finches (*Taeniopygia guttata*). J Comp Psychol 125: 150–161 10.1037/a0020865 21341908

[34] Morton ES (1975) Ecological sources of selection on avian sounds. Am Nat 109: 17–34.

[35] Ey E, Fischer J (2009) The “acoustic adaptation hypothesis”—a review of the evidence from birds, anurans and mammals. Bioacoustics 19: 21–48 10.1080/09524622.2009.9753613

[36] Brumm H, Slater PJB (2006) Animals can vary signal amplitude with receiver distance: evidence from zebra finch song. Anim Behav 72: 699–705 10.1016/j.anbehav.2006.01.020

[37] Boncoraglio G, Saino N (2007) Habitat structure and the evolution of bird song: a meta-analysis of the evidence for the acoustic adaptation hypothesis. Funct Ecol 21: 134–142 10.1111/j.1365-2435.2006.01207.x

[38] Ryan MJ, Kime NM (2003) Selection on long-distance acoustic signals. In: Simmons AM, Popper AN, Fay RR, editors.Acoustic CommunicationvNew York: Springer-Verlag. pp. 225–274.

[39] Ryan MJ, Cocroft RB, Wilczynski W (1990) The role of environmental selection in intraspecific divergence of mate recognition signals in the cricket frog, *Acris crepitans* . Evolution 44: 1869–1872. 28567808 10.1111/j.1558-5646.1990.tb05256.x

